# Numerical Optimization of a Fully Cross-Coupled Rectifier Circuit for Wireless Passive Ultra Low Power Sensor Nodes

**DOI:** 10.3390/s19204527

**Published:** 2019-10-18

**Authors:** Dominik Mair, Manuel Ferdik, Christof Happ, Michael Renzler, Thomas Ussmueller

**Affiliations:** Microelectronics and Implantable Systems Group, Department of Mechatronics, University of Innsbruck, 6020 Innsbruck, Austria

**Keywords:** wireless sensors, rectifier, power conversion efficiency, voltage conversion efficiency, impedance matching, internet of things, radio frequency identification, circuit optimization, conjugate gradient, low-power electronics, wireless power transmission

## Abstract

In the context of the Internet of Things, billions of devices—especially sensors—will be linked together in the next few years. A core component of wireless passive sensor nodes is the rectifier, which has to provide the circuit with sufficient operating voltage. In these devices, the rectifier has to be as energy efficient as possible in order to guarantee an optimal operation. Therefore, a numerical optimization scheme is proposed in this paper, which is able to find a unique optimal solution for an integrated Complementary Metal-Oxide-Semiconductor (CMOS) rectifier circuit with Self-Vth-Cancellation (SVC). An exploration of the parameter space is carried out in order to generate a meaningful target function for enhancing the rectified power for a fixed communication distance. In this paper, a mean conversion efficiency is introduced, which is a more valid target function for optimization than the Voltage Conversion Efficiency (VCE) and the commonly used Power Conversion Efficiency (PCE) and is defined as the arithmetic mean between PCE and VCE. Various trade-offs between output voltage, PCE, VCE and MCE are shown, which provide valuable information for low power rectifier designs. With the proposed method, a rectifier in a low power 55 nm process from Globalfoundries (GF55LPe) is optimized and simulated at −30 dBm input power. A mean PCE of 63.33% and a mean VCE of 63.40% is achieved.

## 1. Introduction

In the context of the Internet of Things (IoT), the number of connected nodes in future scenarios is estimated to be 20, 40 or even 50 billion devices [1,2,3,4]. A large number of these will be sensors, which can be used for gathering information and thus make our environment “intelligent”. This information can also be used for making decisions and controlling actuators. To reduce the effort for installation, wireless sensor nodes will be the method of choice. However, this approach raises the problem of power supply. To reduce the environmental impact of batteries, passive wireless communication systems such as Ultra-High-Frequency (UHF) Radio-Frequency Identification (RFID) are attracting more and more attention [5,6,7,8,9].

The energy for passive sensor nodes has to be harvested from the environment. In systems based on backscattering—such as UHF RFID—the energy for the transponder is taken from an electromagnetic wave, which is provided by a reader. To provide the system with energy, the incident wave has to be rectified after being received by an antenna. To create a sufficient supply voltage from the incident wave, the rectifier has to work as efficiently as possible. The exact design of the rectifier depends on the employed circuitry, used antenna and targeted communication distance.

According to the Friis transmission equation, the available energy decreases quadratically with the distance between the reader and the transponder. Thus, the rectifier’s performance is of utmost importance and can be described using Power Conversion Efficiency (PCE) and Voltage Conversion Efficiency (VCE). These two parameters describe the amount of power and voltage, respectively, which is available at the output of a rectifier for a given input signal, where VCE is defined per stage. Typically, only PCE is considered as a parameter for optimization. However, this leads to several problems for the automated optimization of multistage rectifiers, as is described in the following paragraphs. If VCE is low, more stages are needed to achieve the desired output voltage $V_{\mathrm{out}}$. This leads to increased diode losses in the rectifier, which in turn leads to a drop in the PCE [10]. This means that, when only PCE is optimized, the input voltage increases, to counter the eventual losses. However, an increasing input voltage lowers the modulation depth, due to the RF-limiter, which then limits the input voltage to protect the subsequent circuitry. Thus, both PCE and VCE should be used for optimizing a rectifier.

However, maximizing both parameters simultaneously is not possible, because there are mutual dependencies among PCE, VCE, input impedance and input voltage. A changed input impedance leads to a changed input voltage, which in turn affects the PCE and VCE. When optimizing the efficiency, the geometric and electrical quantities of the rectifier circuit must be changed again, influences the impedance matching. Thus, the rectifier can only be optimized to a limited extent manually for integrated circuits. However, most publications concerning rectifier design do not mention specifics on how the rectifier itself is optimized. Additionally, most publications do not mention a high VCE as a design goal [10,11,12,13,14,15,16,17,18,19]. We show that using only PCE or VCE is not feasible for an automated design process. Thus, this paper presents an automated optimization process and introduces a new parameter called Mean Conversion Efficiency (MCE), defined as the arithmetic mean of PCE and VCE.

The presented procedure is performed on a 55 nm process and implemented using the simulation tool “Cadence”. An integrated circuit design optimization problem is solved by using a derived target function and a suitable optimization method for a multistage fully cross-coupled rectifier, consisting of four transistors and two additional input capacitances per stage. Finally, an optimized circuit is analyzed regarding process and temperature variations by using corner- and Monte Carlo-simulations for an input power of −30 dBm.

## 2. Rectifier Topology

Typical rectifier circuits employ conventional diodes in order to rectify an input voltage. The diode’s main parameters such as on-resistance and reverse leakage current limit the maximum PCE and VCE. Thus, diodes with a small turn-on voltage such as Schottky diodes are favored, especially for ultra low power devices. However, in the context of integrated circuits, these require costly fabrication processing and are highly dependent on temperature. This is why in most applications diode-connected Metal-Oxide-Semiconductor Field-Effect Transistors (MOSFETs) are used. The effective turn-on voltage of these circuits is approximately the threshold voltage of the used MOSFETs. One method for an increased efficiency is to decrease the threshold voltage $V_{\mathrm{th}}$ by gate biasing techniques [20,21,22,23,24]. In the following, various gate biasing techniques are presented and a suitable rectifier topology is derived [20,25].

### 2.1. Gate Biasing

**Figure 1:**
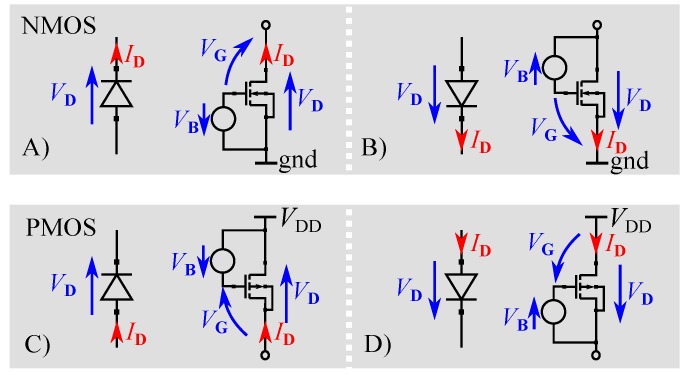
Diode connected NMOS (**A**,**B**) and PMOS (**C**,**D**) for different current directions. The voltage $V_{G}$ is biased by the voltage $V_{B}$. The depicted diodes indicate the equivalent diode operation of the MOSFET circuits.

A standard diode-connected MOSFET features a connection between gate and drain. Instead of a simple connection, gate biasing applies a bias voltage $V_{B}$ between these terminals. The main gate biased transdiode configurations are depicted in Figure 1. Due to the chosen current and voltage directions, as indicated by the arrows in Figure 1, all following statements are valid for the depicted configurations. The gate voltage drop $V_{G}$ can be calculated using:

(1) $$V_{G}=V_{D}+V_{B}$$

The effective turn-on voltage $V_{\mathrm{TO}}$ is defined as the value of $V_{D}$ at which conduction of the diode-connected MOSFET starts to take place. It can be calculated using Equation (1) and setting $V_{G}=V_{\mathrm{th}}$:

(2) $$V_{TO}=V_{th}-V_{B}$$

**Figure 2:**
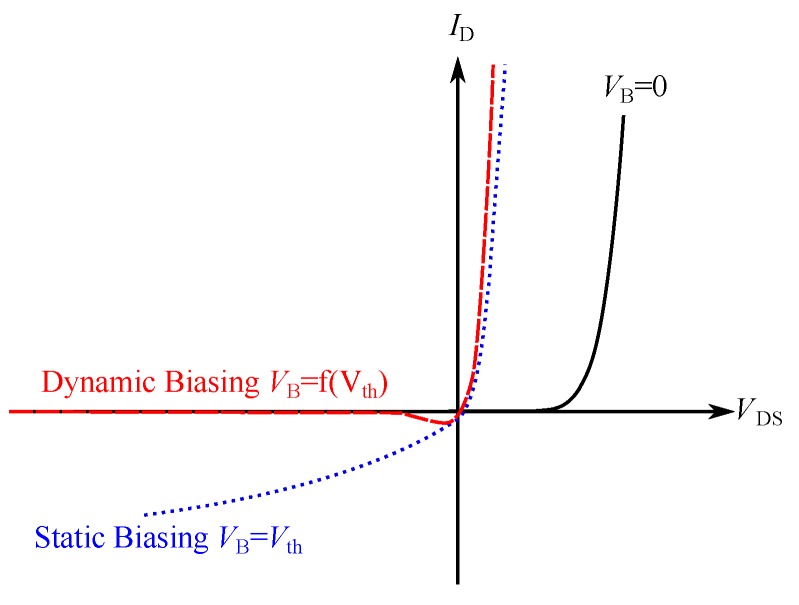
I-V curves of diode-connected MOSFETs (as shown in Figure 1) with different bias voltages.

From this equation, it can be seen that, for $V_{B}=0$, $V_{\mathrm{TO}}$ is equal to $V_{\mathrm{th}}$, which is the turn-on voltage of a non-biased transistor. If a positive voltage $V_{B}>0$ is applied, $V_{TO}$ decreases according to Equation (2). There are two techniques employing this fact for gate biasing [20]:

- Static Gate Biasing: If $V_{B}$ is equal to $V_{th}$, the effective turn-on voltage can be decreased down to zero. Although $V_{TO}=0$ is possible, problems arise for negative values of $V_{D}$. In Figure 2, it can be seen that, for $V_{D}<0$ and $V_{G}=V_{\mathrm{th}}$, reverse currents appear, which lead to losses and are therefore not suitable for low-power devices .
- Dynamic Gate Biasing: To limit reverse currents, dynamic gate biasing varies $V_{B}$ with respect to $V_{D}$. If $V_{D}$ is negative, $V_{B}$ should be low to avoid reverse currents and, if $V_{D}$ is positive, $V_{B}$ should be high in order to minimize the threshold voltage. Figure 2 shows the results of dynamic gate biasing for different values of $V_{B}$.

There are several techniques in order to generate the needed bias voltage for rectifier circuits:

- External-Vth-Cancellation (EVC): Techniques using an external voltage for the gate biasing are called EVC. For passive nodes, an external power supply is not available and thus this technique cannot be used [26].
- Internal-Vth-Cancellation (IVC): IVC generates the biasing voltages using additional circuitry powered by the rectifier. However, similar to EVC, IVC requires additional hardware, which in turn leads to an increased power consumption. This makes this technique not feasible for low power applications [21].
- Self-Vth-Cancellation (SVC): Vth-Cancellation circuits using their self-generated voltages for biasing without additional hardware are called SVC circuits [20].

**Figure 3:**
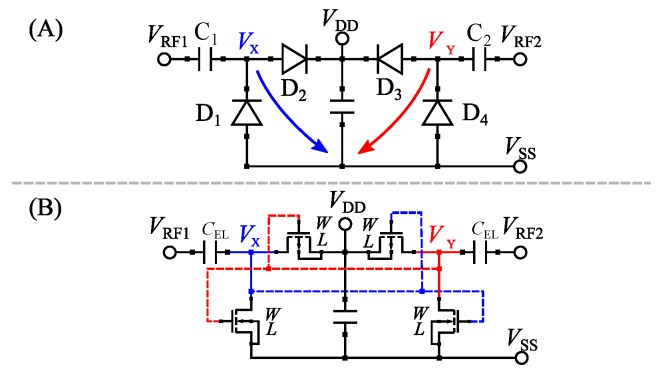
Fully cross-coupled rectifier circuit with diodes (**A**) and MOSFET configuration containing the design parameters (**B**). The gate biasing voltages are generated by the cross-coupled stages (D1–D2 and D3–D4). $V_{\mathrm{RF}1}$ and $V_{\mathrm{RF}2}$ are balanced input signals. The voltages $V_{X}$ and $V_{Y}$ are formed using the input capacitances $C_{1}$, $C_{2}$ and the diode-connected MOSFETs $D_{1}$ and $D_{4}$ (Figure 1B) as well as $D_{2}$ and $D_{3}$ (Figure 1D).

Based on the characteristics mentioned above, dynamic Self-Vth-Cancellation is the preferred method for efficient rectifiers. A widely used topology is the fully cross-coupled rectifier, which is therefore used for the proposed optimization procedure [25,27]. The topology of a fully cross-coupled rectifier is depicted in Figure 3.

### 2.2. Fully Cross-Coupled Rectifier

#### 2.2.1. Basic Operation

If $V_{\mathrm{RF}1}-V_{\mathrm{SS}}$ is negative, the diode D1 is forward biased and capacitance C1 is charged. If $V_{\mathrm{RF}1}-V_{\mathrm{SS}}$ is positive, the diode D1 is reverse biased and the voltage stored on C1 adds up to the input voltage $V_{\mathrm{RF}1}$, resulting in $V_{x}$. The same principle applies to $V_{\mathrm{RF}2}$, C2 and D4, resulting in $V_{y}$. Further rectification with D2 and $C_{\mathrm{out}}$ as well as D3 and $C_{\mathrm{out}}$ results in a DC output voltage equal to $V_{\mathrm{DD}}-V_{\mathrm{SS}}$.

#### 2.2.2. Biasing

The proposed topology uses dynamic gate biasing as described in Section 2.1. If $V_{x}$ is negative, diode D1 is in forward biasing condition and $V_{y}$ is positive. Thus, $V_{y}$ can be used as a biasing voltage $V_{B}$ (Figure 1A) for D1 and, therefore, the effective turn-on voltage is decreased, as explained in Section 2.1. However, if $V_{x}$ is positive, $V_{y}$ is negative, resulting in a decreasing biasing voltage $V_{B}$ for D1 and low reverse currents. The same statements apply for diode D4. The bias voltage $V_{B}$ is equal to $V_{x}$ and forward bias condition is dependent on $V_{y}$.

### 2.3. Design Considerations

For the rectifier design several mutually dependent parameters have to be considered, which renders an analytical approach impossible. The design parameters change the input impedance, thus the matching network or antenna structure has to be adjusted after a parameter adaption. This results in a different input voltage and therefore a change in conversion efficiency. These mutually dependent parameters are explained in the following.

The MOSFETs $D_{1}-D_{4}$ shown in Figure 3 can be adjusted by the design parameters width (*W*) and length (*L*), which have been chosen to be same for all transistors. The values of the capacitors $C_{1}$ and $C_{2}$ are determined by the capacitance edge length ($L_{\mathrm{Cap}}$).

Because the voltages $V_{x}$ and $V_{y}$ are used for biasing, the effectiveness of this rectifier topology depends strongly on the input voltage. Assuming perfect conjugate matching, the Friis transmission equation [28]

(3) $$P_{r}=P_{t}\;D_{t}\;\eta_{t}\;D_{r}\;\eta_{r}\;\underset{1/D_{f}}{\underset{︸}{{\left(\frac{\lambda }{4\;\pi \;d}\right)}^{2}}}$$

can be used to calculate the transponder voltage ($V_{\mathrm{Tag}}=V_{RF1}-V_{RF2}$) amplitude

$${\hat{V}}_{\mathrm{Tag}}=\underset{\frac{\sqrt{2}}{\sqrt{D_{f}}}}{\underset{︸}{\sqrt{2}\left(\frac{\lambda }{4\;\pi \;d}\right)}}\;\underset{\mathrm{Antenna}}{\underset{︸}{\sqrt{D_{t}\;\eta_{t}\;D_{r}\;\eta_{r}}}}\;\underset{\mathrm{Impedance}}{\underset{︸}{\sqrt{\frac{\left(R^{2}+X^{2}\right)}{R}}}}\;\underset{\mathrm{Power}}{\underset{︸}{\sqrt{P_{t}}}}$$

This amplitude depends on its input impedance (R+jX), the free-space path loss $D_{f}$, the antenna directivity and efficiency of transmitting ($D_{t},\eta_{t}$) and receiving antenna ($D_{r},\eta_{r}$) as well as the transmission power ($P_{t}$) of the reader. The analytical calculation of the rectifier’s input impedance is not feasible, thus simulations have to be carried out [29,30,31].

## 3. Concept

**Figure 4:**
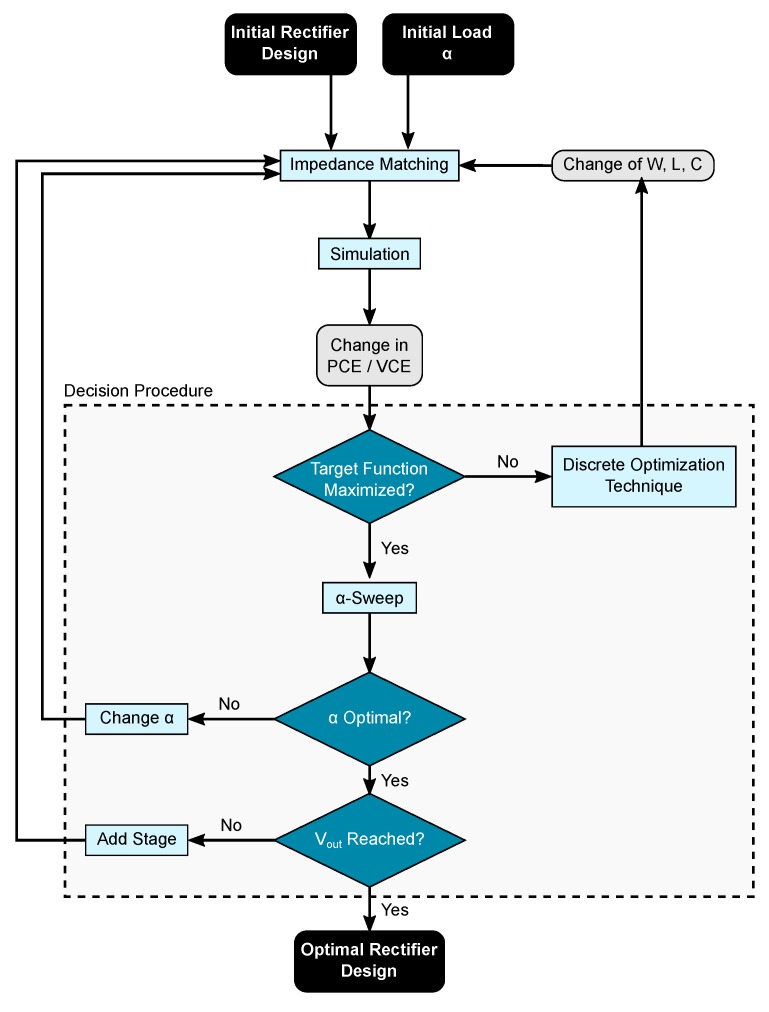
Proposed optimization scheme depicting the different steps. To create the simulation the dependencies of PCE and VCE have to be considered, because the conversion efficiencies strongly depend on the amplitude of the input voltage.

The discussed mutual dependencies of the involved parameters, as well as the infeasibility of analytical calculations, lead to the fact that an iterative optimization process is the method of choice. This section describes the proposed optimization procedure, depicted in Figure 4, step-by-step.

### 3.1. Test Bench

**Figure 5:**
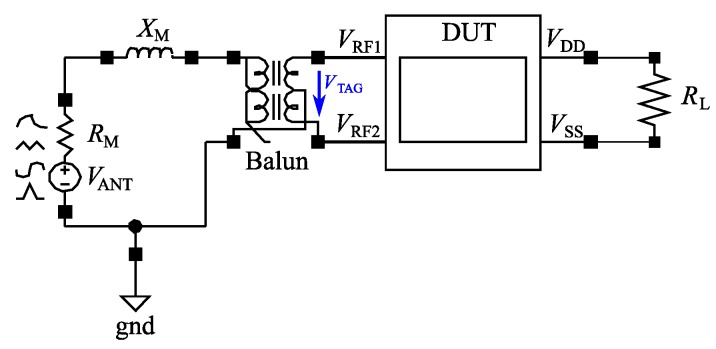
Test bench used for the optimization. The balanced input signals $V_{\mathrm{RF}1}$ and $V_{\mathrm{RF}2}$ are generated by an antenna-equivalent power source with the resistance $R_{M}$ and reactance $X_{M}$ combined with a balun. The rectifier is pictured as a Device Under Test (DUT) .

The core element of the circuit analysis is the simulation of the rectifier. The required test bench is depicted in Figure 5. The antenna is modeled by a power source with inner resistance $R_{M}$ and a serial inductive element with reactance $X_{M}$, which makes conjugate matching possible. The output power is determined by the Friis transmission equation (Equation (3)) at a certain distance. The rectifier itself—labeled as a Device Under Test (DUT)—is connected to the antenna via an ideal balun and to the load. The balun is used to generate the required balanced input signals from an unbalanced signal. The value of the load resistance $R_{L}$ is calculated using

(4) $$R_{L}=\frac{{V_{\mathrm{out}}}^{2}}{P_{r}\;\cdot \;\alpha }$$

With the specified rectified output voltage $V_{\mathrm{out}}$ and the available input power $P_{r}$ calculated from Equation (3), the load resistance is defined. The load scaling factor α is in the range [0,1] and determines how much power is tapped from the rectifier. The produced output voltage is the difference of the node voltages $V_{\mathrm{DD}}$ and $V_{\mathrm{SS}}$.

### 3.2. Impedance Matching

To avoid any influence of the matching quality on the results of the simulation, an impedance matching has to be performed in every iteration after the parameters width, length, capacitance edge length or load factor are changed. First, it is verified if matching applies by simulating the power that is available at the DUT and compares it to the input power that should be available ($P_{r}$). If less than 99% is accessible, the input impedance of the rectifier is simulated and the impedance of the antenna is chosen to be the rectifier’s conjugate. With better matching, a higher input power is achieved and therefore the impedance changes. Thus, another simulation has to be carried out. This process is continued iteratively until matching applies. If more than 99% of the input power are available at the DUT, the impedance of the rectifier is handed onto the next step.

### 3.3. Simulation

The main simulation is started with the impedance values obtained by the previous described matching. The simulation carries out a transient analysis followed by a Fourier transformation. The output of this are the voltage, current and power values of the test bench. Using these values, PCE and the VCE value per stage can be calculated.

### 3.4. Decision Procedure

**Figure 6:**
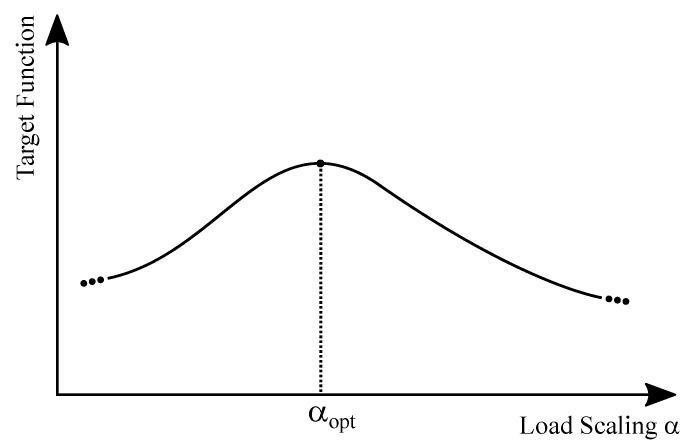
Sweep of the load scaling factor α to obtain the load, at which the target function is at a maximum.

**Figure 7:**
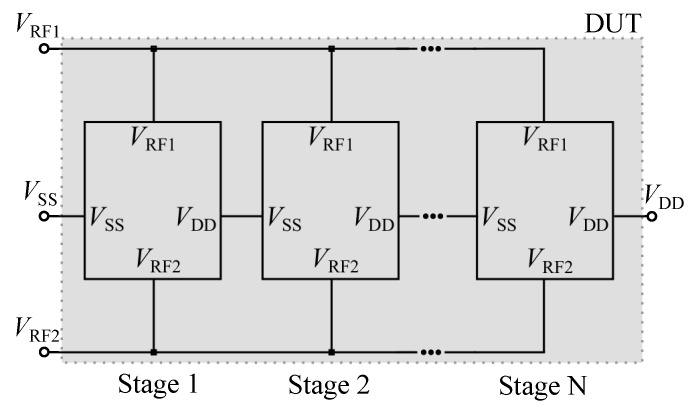
Multistage rectifier stacking scheme. The RF input voltages of every stage are connected together. To achieve higher output voltages, $V_{\mathrm{DD}}$ and the pin of the next stage $V_{\mathrm{SS}}$ are connected. The multistage rectifier with N stages is labeled as a Device Under Test (DUT).

PCE and the VCE are combined into a target function, which is optimized by a discrete optimization technique. If there is no improvement of the target within 10 runs, the target function is satisfied. With better PCE and VCE values, the load scaling factor has to be adjusted in order to guarantee maximum efficiency. This is done by sweeping α in the range of [0,1] and searching for the optimal value $\alpha_{\mathrm{opt}}$ target function is at a maximum, as depicted in Figure 6. If α changes, the simulation procedure starts all over with the impedance matching, otherwise the achieved output voltage $V_{\mathrm{out}}$ has to be analyzed. If the required value has not been reached, another stage is added, as described in Figure 7, and the optimization starts all over. However, if $V_{\mathrm{out}}$ is reached, an optimal rectifier design has been achieved.

## 4. Results

The effectiveness of a rectifier circuit is mainly characterized by the PCE and the VCE per stage. However, no publication takes both parameters into account. Thus, in this section, we show that VCE is indispensable for automatic rectifier design and we propose that the target function has to include both values to achieve a highly efficient design. In addition, an exemplary optimization process is carried out by employing the proposed procedure using the target function derived in this section. Furthermore, we investigate process, mismatch and temperature variations of the optimized rectifier.

### 4.1. Investigation of the Parameter Space

To derive a useful target, an exemplary investigation of the parameter space has to be carried out. The parameters width (*W*), length (*L*) and capacitance edge length ($L_{\mathrm{Cap}}$) are swept for the rectifier circuit. PCE and VCE values are observed. The parameter ranges are determined by the employed manufacturing process and the desired output characteristics of the rectifier and can be seen in Table 1. The system is simulated using the variables shown in Table 2.

**Figure 8:**
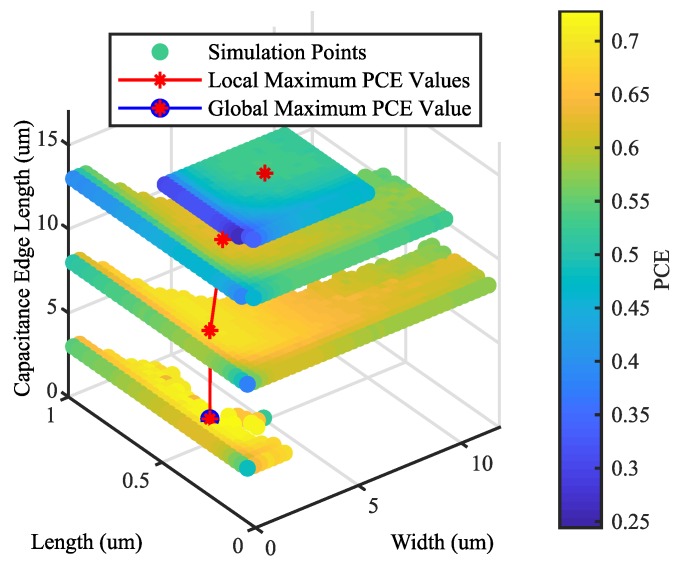
PCE with respect to the parameters width, length and capacitance edge length.

**Figure 9:**
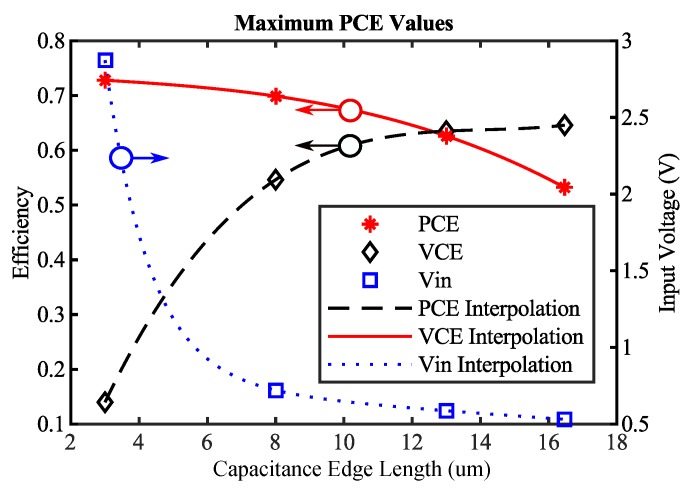
Maximum achievable PCE values for a fixed capacitance and VCE values occurring at the points of maximum PCE. Additionally, the input voltage Vin in dependence of PCE is shown.

In Figure 8, the results of the simulation for PCE are plotted as a function of width, length and capacitance edge length. The maximum achievable PCE is indicated by a red mark for a specific capacitance. The red line shows the optimal path towards the global maximum value, which is indicated by a blue circle. Figure 9 shows the maximum achievable PCE, extracted from Figure 8, as a function of the capacitance edge length. Intermediate points are approximated with least square approximation using third-order polynomials.

The VCE values occurring at the points of maximum PCE are shown in Figure 8. It can be seen that, if only the PCE value is used as an optimization target, VCE decreases. A small VCE value results either in low output voltages, which leads to a higher number of rectifier stages and consequently more diode losses, or in very high input voltages, which is undesirable, as described in the Introduction. Furthermore, depending on the specific process on which the rectifier is manufactured, the input voltage must not exceed a certain value, otherwise the integrated circuit is at risk of being destroyed. In the presented case, the maximum input voltage of the manufactured rectifier is limited to 1.7 V. In typical UHF RFID interfaces, limiting circuits are used to protect the transponder. However, limiters cut of the voltage with a shunt transistor. Thus, the modulation depth of the input RF signal decreases. Another problem is the shunt itself, as it limits the voltage by dissipating the current, thus limiting the maximum output power. Therefore, the PCE alone is not a feasible optimization target.

**Figure 10:**
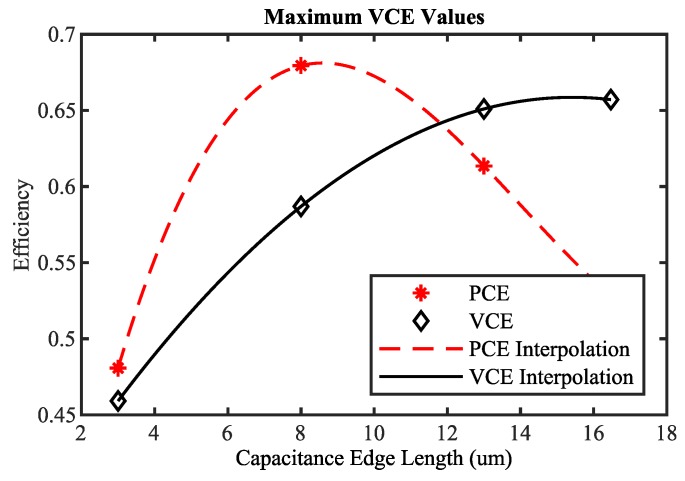
Maximum achievable VCE values per rectifier stage for a fixed capacitance and PCE values occurring at the points of maximum VCE.

The same analysis can be applied to VCE. In Figure 10, the maximum achievable VCE values per rectifier stage for a fixed capacitance and the PCE values occurring at the points of maximum VCE are shown. If only VCE is used for optimization, the PCE value is reduced. However, for low input powers, the PCE has to be as high as possible. In addition, if the VCE value were used for optimization, the derivation of the optimal load scaling factor α would result in an open load ($\alpha_{\mathrm{opt}}=0$). Thus, VCE on its own is also not appropriate as an optimization target.

Based on the presented analysis, it can be seen that PCE and VCE both have to be high in order to guarantee a satisfying rectifier performance. Unfortunately, as shown in Figure 9 and Figure 10, maximum PCE and maximum VCE are not achievable at the same time. Therefore, the arithmetic mean value MCE of PCE and VCE is introduced and investigated as a target function for automatic rectifier optimization.

(5) $$MCE=\frac{PCE+VCE}{2}$$

**Figure 11:**
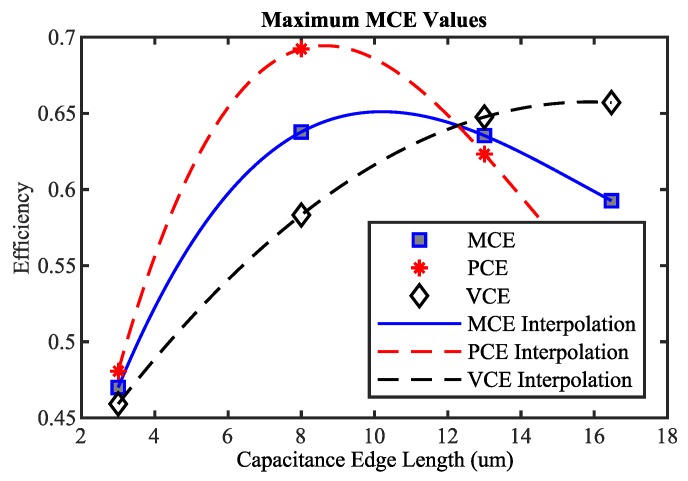
Maximum achievable MCE values per rectifier stage for a fixed capacitance and VCE and PCE values occurring at the points of maximum MCE.

The maximum MCE values for a fixed capacitance are depicted in Figure 11. At the maximum value of MCE, satisfying PCE and VCE values are attained. Furthermore, it can be seen that the optimization goal is a maximization of a concave target function. This is known as a convex optimization problem, where a local maximum corresponds to the global maximum. Local optimization algorithms are therefore the method of choice. A very efficient technique used is the conjugate gradient method, which is widely used and also provided by most integrated circuit simulation tools such as Cadence. Therefore, the proposed optimization scheme is depicted in Figure 4 with MCE as a target function and conjugate gradient as the discrete optimization technique.

### 4.2. Optimization Results

An optimization of a multistage rectifier is carried out. The defined specifications for the rectifier are seen in Table 2. The procedure optimizes wireless power transmission for a minimum input power of −30 dBm. According to Friis’ equation and by using half wavelength dipoles as receiving and transmitting antenna, this input power corresponds to a maximum transmission distance of 64 m. The minimum output voltage is specified to be 1 V. For the optimization, the typical corner (TT) at a temperature of 27 °C is used.

The optimization produces a rectifier with three stages that are stacked according to Figure 7. The value of $\alpha_{\mathrm{opt}}$ was evaluated to be 0.5, which is equivalent to a load resistance $R_{L}$ of 2 MΩ. The output voltage is 1.18 V and MCE is 67.90% (PCE = 69.6%, VCE = 66.2%). The results of the optimized design parameters are shown in Table 3.

**Figure 12:**
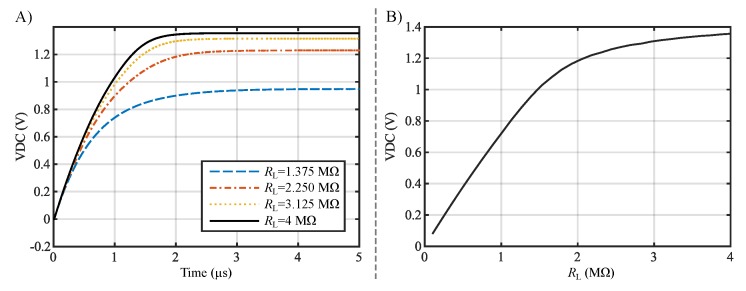
(**A**) Initial transient responses of the rectified output voltage VDC for different load values $R_{L}$ are shown. (**B**) The steady-state voltage $V_{\mathrm{DC}}$ with respect to the load resistance can be seen.

The optimized circuit’s initial transient response for various load resistances $R_{L}$ is depicted in Figure 12A. It can be seen that the steady-state value of the voltage VDC settles more quickly with bigger load resistances and occurs minimum within 4 µs. However, the duration of the settling process also depends largely on the size of the used energy-storage capacitance. For the simulations, it is chosen to be 0.35 pF. In Figure 12B, the steady-state voltages VDC for different loads can be seen. For loads smaller than 1.5 MΩ, the voltage starts to decrease to such an extent that it falls below the minimum specified output voltage of 1 V.

### 4.3. Corner and Monte Carlo Simulations

**Figure 13:**
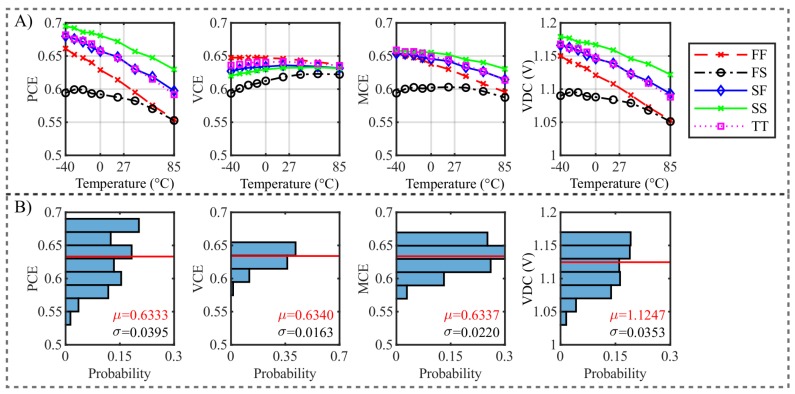
(**A**) Results of post-layout simulations including process (fast–fast (FF), fast–slow (FS), slow–fast (SF), slow–slow (SS), and typical–typical (TT)) and temperature variations ([−40, 85] °C) are depicted. It can be seen that all corners fulfill the minimum specified output voltage of 1 V. (**B**) The results of a post-layout Monte Carlo simulation, including process and mismatch variations in the temperature range of [−40, 85] °C can be seen. The mean values of PCE, VCE, MCE and VDC are 63.33%, 63.40%, 63.37% and 1.1247 V, respectively. It can be seen that the rectifier exceeds the minimum specified output voltage of 1 V within 99.73% of process and mismatch variations.

Another issue is the dependency of the optimized rectifier on process and mismatch variations including parasitics extracted from the layout. Thus, corner and Monte Carlo simulations have to be carried out. The layout is derived from the schematic and the optimization results. All parasitic resistances and capacitances are included in the simulation without any threshold values. The test bench used is the same as employed by the optimization explained in Section 3.1 with an optimal load resistance of 2 MΩ. To consider process variability in circuit design, corner models such as fast–fast (FF), fast–slow (FS), slow–fast (SF), slow–slow (SS) and typical–typical (TT) are used to determine the lower and upper limits of process variation [32]. These corners and temperature variations in [−40, 85] °C are considered. In Figure 13A, the results from corner simulations are depicted. Although the parasitics lead to a non-optimal operation of the rectifier, it can be seen that in all corners the output voltage of the three stage rectifier exceeds the specified voltage of 1 V. In addition, the temperature dependence of the rectifier is interesting. In all corners, a lower temperature indicates also a higher efficiency until this trend starts to reverse close to −40 °C.

Corner simulations are useful for designing a circuit with low simulation time, however they have a few disadvantages. On the one hand, mismatch variations are not included. On the other hand, corners include unlikely cases, which can lead to overdesign of a circuit [32]. To gain additional information, Monte Carlo simulations are carried out. They are constrained to process variations within 3σ. This means that 99.73% of the process variations should be included for the simulations. In addition, the temperature was shifted between −40 °C and 85 °C. In Figure 13B, normalized histogram plots of a Monte Carlo run are depicted. It can be seen that the mean value of the output voltage is equal to 1.12 V with a standard deviation of σ = 35.3 mV. Therefore, the rectifier exceeds the minimum output voltage of 1 V within process and mismatch variations of 99.73%. The MCE’s mean value is 63.37% with a σ of 2.20%. Thus, it can be seen that the circuit behaves well within all corners and Monte Carlo simulations.

The achieved efficiencies of the optimized rectifier circuit in the present study exceed those reported in the literature, as depicted in Table 4, where $V_{\mathrm{out}}$, PCE and VCE are compared to each other. Although high efficiencies are reported for −22.4 dBm [13], studies at input powers as low as −30 dBm are rare [14]. Thus, this work is compared to various measurements [10,11,12] and simulations [13,14,15,16,17,18,19] at higher input powers.

With the proposed optimization method, comparably high efficiencies are achievable. In addition, the high degree of automation has the potential to result in a significant decrease of development time.

## 5. Conclusions

In this paper, an optimization scheme is proposed, which is able to find a unique optimal solution for a fully cross-coupled rectifier circuit for wireless ultra-low power sensor nodes, taking various mutual dependencies into account. We show that PCE alone is not feasible as an optimization target for automatic rectifier design, and therefore propose the Mean Conversion Efficiency (MCE). An optimization of a three-stage fully cross-coupled rectifier is shown. The resulting circuit is analyzed regarding process and mismatch variations with post-layout corner and Monte Carlo simulations within temperatures from −40 °C to 85 °C. A mean PCE of 63.33% at an input power of −30 dBm is achieved, which is superior to other publications.

The demonstrated method works well for fully cross-coupled rectifiers, which is why attempts will be made to adapt and test it for other RF frontend circuits such as demodulators or limiters and other frequency domains and input powers altogether.

Due to the automated nature of the process and its reproducibility, it is possible to compare different technology nodes concerning their performance, thus rendering decision-making benchmarks possible. Additionally, valuable informations for rectifier designers is provided by depicting various mutual dependencies, especially between PCE and VCE.

The presented approach has the potential to reduce development time significantly, while also improving the performance of the developed circuit.

## Figures and Tables

**Table 1:** Range of design parameters used for the exploration of the parameter space. For four capacitance edge length values, the width and length of a MOSFET are varied for the rectifier depicted in Figure 3.

Parameter	Lower Limit	Upper Limit
Width	150 nm	11.9 µm
Length	60 nm	1 µm
Capacitance Edge Length	3 µm	16.5 µm

**Table 2:** Variables used for the exploration of the parameter space. The input power $P_{r}$ is calculated using Equation (3) with an antenna gain of 2.15 dBi and a transmitting power $P_{t}$ of 2 W.

Variable	Value	Variable	Value
Frequency *f*	865 MHz	Distance *d*	64 m
Load Factor α	0.5	$D_{t}\cdot \eta_{t},D_{r}\cdot \eta_{r}$	2.15 dBi
Specified Voltage $V_{\mathrm{out}}$	1 V	Input Power $P_{r}$	−30 dBm

**Table 3:** Results of an optimization process.

Parameter	Value
Width *W*	1.55 µm
Length *L*	280 nm
Capacitance Edge Length *C*	10.75 µm
Antenna Resistance $R_{M}$	242.3 Ω
Antenna Reactance $X_{M}$	6701.3 Ω

**Table 4:** Comparison of the results from this work (TW) including parasitics to measurements marked with an exclamation mark (!) and simulations reported in the literature. Values marked with an asterisk (*) are estimates based on figures and tables.

Ref.	Frequency	Node	*P*_r_	*V*_out_	PCE	VCE
[10](!)	915 MHz	90 nm	−18.83 dBm	1.2 V	25.0%	-
[11](!)	950 MHz	300 nm	−14.0 dBm	1.5 V	11.0%	-
[12](!)	900 MHz	40 nm	−20.1 dBm	1.0 V	41.4%	-
[13]	900 MHz	-	−22.4 dBm	1.2 V	63%	61%(*)
[14]	953 MHz	180 nm	−30 dBm	1.0 V	20%(*)	-
[15]	915 MHz	55 nm	−17 dBm	2.45 V	60%(*)	-
[16]	866 MHz	Discrete	0 dBm	4.6 V(*)	55%(*)	-
[17]	860 MHz–960 MHz	180 nm	−10 dBm	1.1 V	30%	-
[18]	868 MHz	Discrete	−4 dBm	1.9 V(*)	54%(*)	-
[19]	570 MHz	180 nm	−26 dBm	0.64 V(*)	27%	-
TW	865 MHz	55 nm	−30 dBm	1.12 V	63.33%	63.40%
